# Depression and suicidal ideation in medical students in China: a call for wellness curricula

**DOI:** 10.5116/ijme.52e3.a465

**Published:** 2014-02-15

**Authors:** Kunmi Sobowale, A. Ning Zhou, Jingyi Fan, Ni Liu, Renslow Sherer

**Affiliations:** 1 Pritzker School of Medicine, The University of Chicago, USA; 2 Department of Pediatrics, Zhongnan Hospital of Wuhan University, China; 3 Child/Adolescent Psychiatry, North Shore University Health System, USA; 4 Section of Infectious Disease and Global Health, Department of Medicine, University of Chicago, USA

**Keywords:** China, medical student, depression, suicidal ideation, wellness

## Abstract

**Objectives:**

To investigate rates of depression and suicidal ideation in medical students in mainland China and to explore wellness curricula and mental health services available to students.

**Methods:**

Second and third year medical students (N=348) at one medical school in mainland China completed the Patient Health Questionnaire-9 (PHQ-9). Based on responses to the PHQ-9, students were labeled as depressed, with suicidal ideation, and/or impaired. Additionally, students’ feedback from a focus group (N=30) evaluating the current state of the school’s wellness curricula and mental health services was thematically analyzed.

**Results:**

A total of 348 students responded (response rate = 99%) to the survey. Forty-seven of 348 (13.5%) students had moderate-severe depression. The mean PHQ-9 score was 6.02 (SD=3.44). Seven and a half percent of students reported suicidal ideation. The frequency of depression and suicidal ideation did not differ between second and third year medical students (p = 0.52). Nearly 30% of depressed students reported suicidal ideation. Depression and suicidal ideation were strongly correlated (r = 0.42, p < 0.001). Students with depression (p < 0.0001) or suicidal ideation (p = 0.004) were more likely to be impaired compared to students who were not. Focus group participants reported only off-campus student counseling services available to medical students in distress. No wellness curricula were established.

**Conclusions:**

Rates of depression and suicidal ideation are high in medical students in mainland China. Mental health services are deficient and unlikely to address distress in students. Chinese medical schools should offer mental health support and treatment at an early stage, such as wellness curricula and proactive student counseling.

## Introduction

Recent studies have revealed poor mental health in Chinese physicians.^1^ The frequency of depressive symptoms among Chinese physicians is over 65%, which is a rate much higher than their colleagues worldwide.^2^ Depression is highly associated with burnout in Chinese physicians.^3^ Over 12% of Chinese physicians report burnout, which is a form of physician distress. In fact, the effects of depression extend beyond burnout and other forms of physician distress.^3^ Depressed physicians provide lower quality patient care and more commonly commit medical errors.^4^ Focusing on the causes of distress, particularly depression and its consequences, in Chinese physicians is important. Since physician distress is hypothesized to begin in medical school, medical school presents an excellent opportunity to avert depression via wellness curricula and mental health programming.Medical students have higher levels of depressive symptoms than the general population.^5^ Depressed medical students are more likely to experience burnout or drop out of medical school.^6^^,^^7^ Additionally, depressed students are more likely to consider or commit suicide.^8^^-^^10^ Recent studies find 11.2-17.4% of medical students experience suicidal ideation annually.^6^^,^^11^ Many factors, including academic stress and workload,^12^^,^^13^ student abuse and hazing,^14^^,^^15^ and sleep deprivation,^16^ have been attributed to the increased vulnerability in medical students to depression. Medical students in mainland China experience additional, unique stressors. For example, a competitive job market and high incidence of violence against physicians may contribute to increased levels of stress in Chinese medical students.^17^^,^^18^Due to these additional stressors, one would expect Chinese medical students to suffer from high levels of depressive symptoms and suicidal ideation. However, little is known about depression and suicidal ideation in medical students in mainland China. One recent study in mainland China found that 16.8% students were depressed.^19^ In addition to depression, suicidal ideation is a strong predictor of attempted and committed suicide. Yet only one published study, conducted in Taiwan, investigated suicidal ideation in the Chinese medical student population.^20^ In the Taiwanese study, levels of suicidal ideation were high (i.e., over 10%). Given the limited amount of research conducted, more information on depression and suicidal ideation in medical students in mainland China is necessary.However, solely detecting the presence of mental illness in Chinese medical students is unlikely to improve well-being. Even when diagnosed with mental illness, many medical students often do not receive care.^21^ Less than 10% of depressed medical students seek help.^22^ Lack of treatment can lead to poorer outcomes in medical students.^21^ Interventions are required to prevent mental illness in medical students and to provide care when mental illness is present. To this end, a number of medical schools in the US and Canada have developed wellness programs to improve student well-being.^23^^-^^25^ Such wellness programs can be reactive or proactive.^23^Reactive wellness programs include student counseling centers and are used when a student is in acute distress. Proactive wellness programs promote mental health by providing techniques to manage stress, coordinating support from peers and faculty, and empowering students to practice self-care to prevent mental illness. One example of an established medical student wellness program exists at Vanderbilt School of Medicine.^23^ Wellness at Vanderbilt is focused on three key areas: mentoring and advising through an advisory college, student leadership through a student wellness committee, and personal growth through a longitudinal curriculum focusing on the personal development of medical students.^23^ Neither study of mental illness in medical students in mainland China nor Taiwan^20^ reported the availability of wellness programs and mental health services to students. Because of the potential beneficial effects of wellness programs and mental health services, their availability on Chinese medical campuses warrants study.Given the negative consequences of poor mental health on medical students and their future careers, identification of mental illnesses afflicting Chinese medical students and the available services to improve their well-being is important. Therefore, we measured rates of depression and suicidal ideation among medical students at one medical school in mainland China. In addition, we assessed the existence of wellness curricula and available mental health services for medical students in the same setting.

## Methods

### Participants

A cross-sectional study was conducted at a medical school in mainland China in 2012. Three hundred forty-eight preclinical second and third year Chinese medical students from two medical campuses were surveyed. The medical students received no reward for taking part in the study. The surveys were anonymous with the exception of class year.We invited 30 students from the third year medical student class to participate in a focus group. We inquired about existing wellness programs and mental health resources available at the medical school. Drawing on the established structure of wellness programming at Vanderbilt School of Medicine described above, we inquired about the existence of formal mentoring and advising from faculty and upper class medical students, student organizations focused on wellness, and dedicated curricula designed by the office of medical education to address wellness.^23^ Finally, we asked students about facilities available to students experiencing emotional distress or mental illness. This study was approved by Wuhan University’s Institutional Review Board.

### Instruments

We administered the Patient Health Questionnaire-9 (PHQ-9). The PHQ-9 is a validated tool used to screen for depression based on DSM-IV criteria.^26^ The PHQ-9 has been used in the Chinese population with good reliability (Cronbach’s alpha=0.857) and with a sensitivity and specificity of 91% and 97%, respectively.^27^ The PHQ-9 is a nine-item self-reported scale that assesses the severity of a number of depressive symptoms over the past two weeks from zero (i.e., not at all) to three (i.e., nearly every day). The total score ranges from 0 to 27, subdivided into five categories: 1 to 4 is minimal, 5 to 9 is mild, 10 to 14 is moderate, 15 to 19 is moderate–severe; and 20 and above is severe.^26^ Item nine of the scale assesses suicidal ideation using the phrase “thoughts that you would be better off dead or hurting yourself.” Impairment was assessed by the PHQ-9 single item “how difficult have these problems made it for you to do your work, take care of things at home, or get along with other people?” Positive findings suggest a level of impairment caused by depressive symptoms.

### Data analysis

The independent variable was class year, and the dependent variables were depression, suicidal ideation, and impairment. Class year was coded as a dichotomous variable (i.e., second year vs. third year). Consistent with the prior literature, students with a PHQ-9 score of 10 or greater were labeled depressed.^26^^,^^28^ Students answering positively to item nine or the impairment item were labeled, respectively, as having suicidal ideation and/or impairment. Chi square analysis was used to investigate association between class year and depression, suicidal ideation, and impairment, as well as any association between the dependent variables. The Fisher exact test was used when n<5. An alpha level was set at 0.05 for all statistical analysis. The results were analyzed using STATA 11.0.^29^ The focus group session was translated and recorded in English. Two authors (AZ and KS) reviewed the focus group transcript with application of the Vanderbilt wellness model themes of formal mentorship and advising, student organizations focused on wellness, and dedicated medical school curricula to address student wellness for coding. Any thematic disagreements were settled. From these constructs a coherent narrative was produced.

## Results

### Respondents

A total of 348 students responded to the survey, constituting a response rate of 99%. Three hundred forty-eight students responded to the depression and suicidal ideation questions, and 314 students responded to the impairment question.

### Depression

The mean PHQ-9 score was 6.02 (SD=3.44). Of the 348 total respondents, 122 (35.1%) had no to minimal depression, 179 (51.4%) had mild depression, and 47 respondents (13.5%) had moderate-severe depression (Table 1). No significant differences existed between grade levels in depression score (χ^2^=0.87, 95%CI [-6.8-25.6], p=0.52) see Table 1. Depression was strongly correlated with suicidal ideation (r=0.42, p<0.0001) and impairment (r=0.43, p=<0.001). Nearly 28% of the depressed students reported suicidal ideation, compared to 4.3% of the non-depressed students (Fischer, 95%CI [19.9–59.0], p < 0.0001) see Table 3. Ninety- three percent of the depressed students reported impairment, compared to the 55.4% of non-depressed students (χ^2^=20.0, 95%CI [17.2–23.4], p<0.0001) see Table 4.

**Table 1:** Depression, suicidal Ideation, and impairment in medical students in mainland China in 2012 (N=348)

Measure	Total	Grade 2	Grade 3	p	95% CI	
	n(%)	n(%)	n(%)		Lower	Upper
	N=348	N=140	N=208			
PHQ-9 Score						
None to Minimal (Range, 0-4)	122(35)	53(38)	69(33)	0.5	-6.8*	25.6*
Mild (Range, 5-9)	179(51)	71(51)	108(52)			
Moderate to Severe (Range, 10-27)	47(14)	16(11)	31(15)			
Suicidal Ideation	26(8)	10(7)	16(8)	0.8	-17.5**	21.4**
Impairment	189(54)	81(63)	108(58)	0.4	-16.1^†^	4.6^†^

*between moderately to severely depressed and no or minimally depressed individuals

**between individuals with and without suicidal ideation

^†^between individuals with and without impairment

**Table 3:** Suicidal ideation in medical students with and without depression and impairment in mainland China in 2012 (N=348 and N=314 respectively)

Variable	SI* = 0	SI* > 0	p	95% CI	
	n(%)	n(%)		Lower	Upper
By PHQ Score					
PHQ** <10	288(96)	13(4)	< 0.0001	19.9^†^	59.0^†^
PHQ** ≥10	34(72)	13(28)			
By Impairment					
Not impaired	122(98)	3(2)	0.004	15.2^‡^	44.0^‡^
Impaired	168(89)	21(11)			

*Suicidal ideation (SI = 0: no; SI > 0: with)

**Patient Health Questionnaire-9 (≥10=depressed; <10=not depressed)

^†^Students endorsing suicidal ideation with and without depression

^‡^ Students endorsing suicidal ideation with and without impairment

**Table 4:** Impairment in medical students with and without depression and suicidal ideation in mainland China in 2012 (N=314)

Variable	Not Impaired	Impaired	p	95% CI	
	n(%)	n(%)		Lower	Upper
By PHQ Score					
PHQ* < 10	122(45)	152(55)	< 0.0001	17.2^†^	23.4^†^
PHQ* ≥ 10	3(8)	37(93)			
By Suicidal Ideation					
SI** = 0	122(42)	168(58)	0.004	8.7^‡^	13.9^‡^
SI**> 0	3(13)	21(88)			

* Patient Health Questionnaire-9 (≥10=depressed; <10=not depressed)

**Suicidal ideation (SI=0: no; SI >0: with)

^†^ impaired students with and without depression

^‡^ impaired students with and without suicidal ideation

### Suicidal ideation

Twenty-six respondents (7.5%) indicated the presence of suicidal ideation. Suicidal ideation was strongly correlated with depression (r=0.42, p < 0.0001) and weakly correlated with impairment (r=0.16, p <0.001). Fifty percent of the students with suicidal ideation reported depression, compared to 10.6% of students who had no suicidal ideation (χ^2^=32, 95%CI [10.3–36.3, p=<0.0001) see Table 2. Eighty- eight percent of students with suicidal ideation reported impairment, compared to 57.9% of students with no suicidal ideation (Fisher=0.004, 95%CI [8.7–13.9], p=0.004] see Table 4.

**Table 2:** Depression in medical students with and without suicidal ideation and impairment in mainland China in 2012 (N=348 and N=314 respectively)

Variable	PHQ*<10	PHQ* ≥10	p	95% CI	
	n(%)	n(%)		Lower	Upper
By Suicidal Ideation					
SI** = 0	288(89)	34(11)	< 0.0001	10.3^†^	36.3^†^
SI** > 0	13(50)	13(50)			
By Impairment					
Not impaired	122(98)	3(2)	<0.0001	30.3^‡^	48.5^‡^
Impaired	152(80)	37(20)			

* Patient Health Questionnaire-9 (<10=not depressed; ≥10=depressed)

**Suicidal Ideation (SI=0: no; SI >0: with)

^†^ Depressed students with and without suicidal ideation

^‡^ Depressed students with and without impairment

### Impairment

One hundred eighty-nine respondents (54.3%) indicated some level of impairment (Table 1). 19.6% of students who reported impairment also reported depression, compared to 2.4% of students who reported no impairment (Fisher=< 0.001, 95%CI [30.3–48.5], p<0.0001) see Table 2. Eleven percent of students with suicidal ideation reported impairment, compared to 2.4% of students who reported no impairment (Fisher=0.004, 95%CI [15.2–44.0], p=0.004) see Table 3.

### Wellness and mental health services

Conversations with a student focus group (n=30) at the medical school in China revealed little to no formal wellness curricula at the medical school in the three main domains of wellness described by the Vanderbilt School of Medicine:^23^ formal mentoring and advising, student organizations focused on wellness, and dedicated medical school curricula to address student wellness.

### Formal mentorship and advising

Students reported no formal advising or mentoring system involving faculty or upper class medical students, and remarked that contacting faculty was extremely difficult. One participant said:

“We have no teaching assistants or career advising mentors. In fact, we have no way to contact teachers. Our relationship with teachers is very traditional and not conversational.”

### Student organizations focused on wellness

No student wellness organizations were identified. In fact, student organizations were discouraged, with the exception of the student government and the English club. Moreover, a common theme was lack of time for students to engage in extracurricular activities because of their heavy academic class load. One participant said:

“We have very little freedom. There are a lot of programs that we would be interested in. However, we do not have time to attend events we are interested in.”

### Dedicated medical school curricula to address student wellness

No dedicated wellness curricula were present at the medical school. One participant said:

“Our school does not have any wellness programming that I know of.”

### Facilities available to students experiencing emotional distress or mental illness

Students in distress were offered the opportunity to meet with counselors at the student counseling center. The student counseling center is located on the main university campus, approximately two miles from the medical campus.

## Discussion

We aimed to determine the levels of depression and suicidal ideation and identify wellness resources for medical students. The levels of depression in Chinese medical students were high. The frequency of moderate-severe depression in this sample is comparable to recent studies of medical students in China and the US.^10^^,^^19^^,^^21^^,^^30^ The occurrence of depression was not significantly different between second and third year students. This finding contrasts with previous studies that found depression prevalence increases as medical school progresses.^31^^,^^32^ In China, the third year of medical school is more time-consuming than the second year because students focus on clinically-relevant and case-based classes before clinical rotations begin in the fourth year. Therefore, one might expect higher levels of depression in third year medical students compared to second year students. While our results do not support this tendency, a wider sample containing preclinical students (first, second and third years) and clinical students (fourth and fifth years) might demonstrate this trend.

Another major finding of this study is that 7.5% of students had suicidal ideation during a two-week period. This frequency of suicidal ideation is quite high, slightly higher than the two week frequency reported by Goebert and colleagues for medical students in the US.^30^ However, this frequency is lower than the frequency reported for students in Taiwan.^20^ The same Taiwanese study found an association between depression and suicidal ideation.^20^ Our findings demonstrate that this association also exists in medical students in mainland China. Thus, medical educators, counselors, and providers should assess for suicidal ideation, plans, or past attempts when medical students present with depressive symptoms.Interestingly, the impairment level, the self-report item of interference of symptoms in activities, is strongly correlated with depression score on the PHQ-9 and weakly correlated with suicidal ideation. Impairment in daily activities is a criterion in the diagnosis of many mental illnesses. However, impairment is rarely reported in studies using the PHQ-9. Our findings suggest impairment is an important indicator of students’ mental health and should be reported in subsequent studies using the PHQ-9. The effect impairment has on clinical and academic performance is unclear and subsequent research in this area, both in China and in the United States, would be fruitful.To foster well-being, more accessible outlets of care are necessary for Chinese medical students. In this study, only one counseling center, located approximately two miles from the medical campus, was available to medical students. Indeed, on many Chinese medical campuses, counseling is the sole service available to students in distress.^33^ Given that lack of time is a deterrent to seeking help in medical students, counseling services on the medical campus may increase utilization of care. However, other factors, such as stigma, may explain why medical students do not seek care, and multiple studies have found that medical students are unlikely to utilize student counseling.^21^^,^^22^^,^^34^Wellness curricula may help to alleviate distress in Chinese medical students. However, we found no evidence of wellness curricula on the medical campus we surveyed; wellness curricula have yet to be incorporated in Chinese medical education.^33^ We agree that proactive wellness curricula as opposed to traditional reactive counseling may provide more benefit. These curricula may address the psychological stress from many challenges facing Chinese medical students. One example is the stress of finding a job in mainland China because of the very competitive marketplace.^17^ A wellness program offering occupational advising and mentoring services could provide students information to make this process more manageable. In addition, focusing on interpersonal skills and professional development has the potential to improve the strained doctor-patient relationship in China.^1^ A recent pilot study of a wellness intervention, focusing on coping skills, at a medical university in China found decreased anxiety and depressive symptoms and improved self-esteem after its administration.^33^ Such wellness interventions are necessary to ingrain healthy behaviors and practices that students can apply as physicians.

### Future directions

A thorough needs assessment should be performed to better understand the specific stressors and mental health needs of Chinese medical students. In addition, further study of the impact of a formalized wellness curriculum should be conducted in Chinese medical educational settings, including structured mentoring from faculty and upper classmen, the creation of a student wellness committee, and administration-run wellness programming aimed to improve medical student emotional well-being, resiliency, and sense of commitment to the field of medicine.

### The limitations of the study

This study has several limitations. The study was cross-sectional in nature, so temporal stressors may have influenced students’ responses. The study was conducted in the spring, which is not a particularly stressful academic time for medical students at the medical school. While the surveys were anonymous, students may have felt reluctant to report depressive feelings or suicidal thoughts, leading to underreporting. Additionally, since only medical students at one medical school were surveyed, the results cannot be widely generalized. However, our large sample size and high response rate support the validity of the findings of high depression and suicidal ideation.

## Conclusion

The study results show that medical students in mainland China have high levels of depression and suicidal ideation. Medical schools should make an effort early on to recognize excessive stress and depression among medical students, with particular attention to suicidal ideation and suicide attempts. In order to support the well-being of students, further study and evaluation of new programs outside of counseling services, such as formalized wellness curricula as a component of medical education, are necessary. Wellness programs for medical students have been proven to foster the development and maintenance of the gratifying qualities of medical professionalism in other countries and cultures, and a preliminary study in China has shown a similar outcome. Chinese medical schools should incorporate wellness curricula to provide medical students with the help and resources necessary to become healthy physicians.

### Conflict of Interest

The authors declare that they have no conflict of interest.
